# The association between illness representations, coping resources, and subjective well-being among family caregivers of prolonged mechanical ventilation patients

**DOI:** 10.1080/21642850.2026.2700054

**Published:** 2026-08-31

**Authors:** Tanya Lavrinenko, Shiri Shinan-Altman

**Affiliations:** a Louis and Gabi Weisfeld School of Social Work, Bar-Ilan University, Ramat Gan, Israel

**Keywords:** Prolonged mechanical ventilation, family caregivers, subjective well-being, illness representations, coping resources

## Abstract

**Background:** Prolonged mechanical ventilation (PMV) imposes emotional, physical, and social burdens on family caregivers, yet limited research focuses on their subjective well-being (SWB). This study aimed to explore the factors associated with SWB among family caregivers of hospitalized PMV patients, guided by the Self-Regulation Model.

**Methods:** A total of 134 family caregivers completed questionnaires assessing SWB, illness representations, sense of coherence, and perceived social support.

**Results:** Path analysis revealed that positive illness representations—such as perceptions of shorter illness duration and effective treatment control—enhanced SWB through higher sense of coherence and perceived social support. Conversely, negative representations, including severe perceived consequences and heightened emotional responses, adversely affected SWB. Symptom burden and illness understanding were not significantly linked to coping resources or SWB.

**Conclusions:** Although we offer a mediation model with cross-sectional data, the findings underscore the role of coping resources as mediators in the relationships between illness representations and SWB. Future longitudinal research is recommended to further support the mediation model suggested in our study. Interventions fostering adaptive illness representations and bolstering coping resources, such as sense of coherence and social support, are recommended to alleviate caregiver burden and improve well-being. These insights offer practical guidance for healthcare providers in supporting caregivers and enhancing outcomes for both patients and families.

## Introduction

Technological advances and increased life expectancy have led to more patients needing prolonged mechanical ventilation (PMV) (Jang & Wang, 2020). These patients, often with chronic critical illnesses, typically require mechanical support for over 21 days via endotracheal or tracheostomy tubes (Trudzinski et al., 2022). Those who cannot be weaned usually shift to tracheostomy ventilation and may be transferred to specialised rehabilitation centres to improve outcomes, facilitate home discharge, and reduce intensive care unit (ICU) costs (Verceles et al., 2018). Prognosis for these patients is typically poor, characterised by high morbidity, diminished quality of life, and increased mortality rates (Jang & Wang, 2020). Given their complex medical needs, they rely extensively on primary family caregivers for daily support (Chu et al., 2022).

Caring for a family member receiving PMV is associated with significant changes in the primary caregiver’s daily life (Marcus et al., 2023). For example, it was found that caregivers of PMV patients face considerable physical and psychological challenges including disruptions in sleep, energy, nutrition, and body weight due to prolonged ICU stays (Dale et al., 2020). The burden experienced by these caregivers affects emotional, cognitive, somatic, and social aspects of their lives, which intensify during the patient's hospitalisation (Lee et al., 2020).

When a family member receiving PMV is hospitalised, it can become an overwhelming and traumatic experience for primary caregivers, exacerbating the significant emotional and physical burdens they already endure (Chu et al., 2022; Dale et al., 2020). Caregivers of PMV patients frequently face heightened stress, driven by the suddenness of the hospitalisation and the critical nature of the patient’s condition (Liu et al., 2017; Tabootwong & Kiwannuka, 2020). This stress is associated with heightened feelings of vulnerability and strain and with lower overall well-being, and is linked to long-term exhaustion (Jacobs et al., 2021; Milton, 2021).

*Subjective well-being* (SWB) refers to an individual's overall life satisfaction, encompassing dimensions such as positive affect, personal growth, and autonomy (Diener et al., 1999). This construct includes both positive emotions (e.g. joy, contentment) and negative emotions (e.g. stress, worry) (Diener et al., 1999). Research has shown that, overall, caregiving negatively affects caregivers' health, life satisfaction, and overall liveliness, which are inversely related to their SWB (Milton et al., 2022). However, the SWB of family caregivers for hospitalised patients receiving PMV remains largely understudied. Gaining insight into their SWB is crucial for promoting their resilience and ensuring the sustained welfare of the patients for whom they care. The aim of the current study is to address this gap by exploring factors associated with SWB among these caregivers through the lens of the Self-Regulation Model (SRM) (Leventhal, 1980).

The SRM (Leventhal, 1980) suggests that health outcomes are shaped by individuals' beliefs and perceptions about medical conditions, coping resources, and the consequences of illness. This model explains the formation of cognitive and emotional representations of health threats, with the cognitive component including consequences, timeline, self-control, treatment control, identity, concern, and illness coherence. The emotional component (emotional representations) captures individuals’ emotional responses in relation to the illness. By analysing these perceptions, the SRM offers insights into how caregivers’ perceptions and management of their family member's condition are associated with their SWB.

According to the SRM, illness representations are linked to coping strategies, which may mediate the relationship between illness representations and health outcomes (Leventhal, 1980). However, the mediating role of coping strategies in this process has been questioned in some studies (Cameron & Gignac, 2018; Liu et al., 2021). To deepen the understanding of the SRM, in the current research we shifted the focus from coping strategies to coping resources, exploring whether these resources play a primary role in shaping health outcomes (Shinan-Altman & Afuta-Goldstein, 2020). Coping resources, such as personality traits and support, help manage health threats (Shinan-Altman & Katzav, 2021). In this study we specifically examined two key coping resources: sense of coherence (an internal resource) and perceived social support (an external resource). Prior research has consistently demonstrated the association between these resources and SWB among family caregivers (e.g. Del-Pino-Casado et al., 2019; Sultan et al., 2018).

*Sense of coherence* refers to individuals' ability to perceive life as comprehensible, manageable, and meaningful, equipping them to better cope with stress and maintain their health (Antonovsky, 1987). This construct serves as a protective factor for caregivers, buffering them against the negative consequences of caregiver burden (Gonçalves-Pereira et al., 2021). A meta-analysis underscored the critical role of a strong sense of coherence in promoting caregiver well-being, as it helps mitigate psychological distress and reduce caregiver burden (Del-Pino-Casado et al., 2019). Furthermore, a study among caregivers of individuals with dementia found that illness representations were associated with caregivers’ sense of coherence, with more threatening perceptions linked to a lower sense of coherence (Lo Sterzo & Orgeta, 2017).

*Perceived social support* refers to individuals’ sense of being cared for, valued, and integrated into a network of people concerned with their well-being (Taylor, 2011). This support typically comes from three key sources: family, friends, and significant others (Zimet et al., 1988). According to the SRM, illness representations play a crucial role in shaping social support in two ways (Giannousi et al., 2023). First, caregiving family members' perceptions of their relative’s illness shape their expectations and assessments of the support they receive. These perceptions are associated with the extent to which caregivers seek and accept support (Kalolo et al., 2023). Second, illness representations can also shape the type and quality of support provided by others, as caregivers and social networks may adjust their level of involvement on the basis of how they perceive the severity and manageability of the illness (Cipolletta et al., 2020).

In this study we sought to examine a comprehensive model that integrates the interrelationships among illness representations, coping resources, and SWB in family caregivers of PMV patients during hospitalisation. Additionally, we investigated indirect relationships, with illness representations as independent variables, sense of coherence and perceived social support as mediators, and SWB as the outcome variable. This model is theory driven (Leventhal, 1980) with the awareness that the current study has a cross-sectional design, and only future longitudinal research will be able to fully examine it. Still, studying the interplay between illness representations, coping resources, and SWB among family caregivers of PMV patients is crucial, as such research holds the potential to drive targeted interventions that significantly enhance caregiver resilience, alleviate stress, and profoundly improve both caregiver and patient outcomes.

## Method

### Procedure and participants

Before initiating the study, authorisation was received from the Ethics Committee of Bar Ilan University (approval No. 112101). Participants completed the questionnaires via email, which were administered through the Qualtrics online platform (www.qualtrics.com). Invitations to participate in the survey, focusing on the mental well-being of primary caregivers of PMV patients, were distributed online. Recruitment efforts primarily targeted internet forums and websites dedicated to chronic ventilation, as well as social media platforms (e.g. Facebook pages related to chronic ventilation), enabling access to a wider group of family members of PMV patients. Anonymity was preserved by not requesting any identifying information from participants.

The sample size was calculated with G*Power (ver.3.1) (Faul et al., 2009). Considering a power of 0.85, an alpha level of 0.05, and a moderate effect size of *f* ^2^ = 0.15, a regression with 10 predictors would require 131 participants. For a mediation effect (Kenny, 2017), given a minimum *β* of 0.27 for the direct associations, alpha = 0.05, and a power of 0.80, the required minimum sample size for the indirect effect would be 133 participants. Study inclusion criteria were family members who were primary caregivers (children and spouses) of PMV patients in a medical setting. Exclusion criteria were minors (under the age of 18); providing responses to the items in a similar pattern (e.g. choosing the same answer across multiple consecutive items or within the whole questionnaire); or not completing the questionnaire in its entirety (*n* = 15). Measures were taken to avoid having participants respond multiple times, such as using the ‘Prevent Ballot Box Stuffing’ feature on Qualtrics. In total, the study included 134 main caregivers of PMV patients.

### Measures

#### Dependent variable

*Subjective well-being* was assessed via the 14-item Mental Health Continuum-Short Form (MHC-SF) (Lamers et al., 2011). Participants rated the frequency of their well-being and distress experiences over the past month on a 6-point Likert scale, ranging from 1 (*never*) to 6 (*every day*) (e.g. ‘In the past month, how often did you feel happy?’). Mean scores were computed, with higher scores indicating greater levels of SWB (Cronbach's alpha = 0.91).

#### Independent variables

*Illness representations* were assessed via the Brief Illness Perception Questionnaire (Benyamini & Shiloh, 2011; Broadbent et al., 2006) which evaluates the components of illness perception as outlined by the SRM (Cameron & Leventhal, 2003). This assessment encompasses a single item for each of eight dimensions, and for the current study was adapted to the family caregivers rather than the patients: the consequences of the disease on various life domains as perceived by the caregivers; the disease's timeline (chronic vs. acute); the degree of control caregivers feel they have over the patient’s disease (self-control); the degree of control caregivers perceive they have over the treatment the patient is receiving (treatment control); the burden of symptoms among the caregivers (symptom burden); caregivers’ level of concern regarding the disease (concern); caregivers’ understanding of the disease (understanding); and the emotional representations of the illness among the caregivers (e.g. ‘To what extent do you feel that you understand your family member’s medical condition?’). Each dimension was measured on a Likert-type scale ranging from 0 (*low rating*) to 10 (*high rating*).

*Sense of coherence* was measured using the SOC-13 questionnaire (Eriksson & Mittelmark, 2017). The SOC-13 has been validated worldwide in different languages including English (Rohner et al., 2022), and was translated into Hebrew and validated by Bernstein and Carmel (1987). Participants rated each statement on a 7-point Likert-type scale, ranging from 1 (*never*) to 7 (*always*) (e.g. ‘Do you have very mixed-up feelings and ideas?’). Mean scores were computed, with higher scores indicating greater levels of sense of coherence (Cronbach's alpha = 0.88).

*Perceived social support* was assessed through the Multidimensional Scale of Perceived Social Support (MSPSS) (Zimet et al., 1988), which is based on a 12-item Likert scale designed to capture perceived social support across three distinct domains: family, friends, and significant others (e.g. ‘I can count on my friends when things go wrong’). Participants were asked to rate their agreement or disagreement with each statement on a 7-point Likert-type scale, ranging from 1 (*strongly disagree*) to 7 (*strongly agree*). Mean scores were computed, with higher scores denoting greater levels of perceived social support (Cronbach's alpha = 0.89).

*Sociodemographic details* included age, gender (female/male), marital status, religiousness, education, kinship to a ventilated family member, and living with a ventilated member before hospitalisation (yes/no). Additionally, participants were required to indicate that their family member was receiving PMV and was currently hospitalised, providing details about the duration of hospitalisation for receiving PMV, the family member's age, gender, and any additional chronic illnesses.

#### Data analysis

Data were analysed using SPSS ver. 29. Descriptive statistics were utilised for the participants’ demographic details and the study variables. As SWB, sense of coherence, and perceived social support were negatively skewed (skewness = −1.00 to −2.34, *SE* = 0.21), they were exponentially transformed. The illness representation of self-control was positively skewed (skewness = 1.80, *SE* = 0.21) and was thus logarithmically transformed. Associations between the study variables were examined with Pearson correlations, using the following categorisation: 0–0.20 indicating weak, 0.21–0.50 indicating moderate, 0.51–0.80 indicating high, and 0.81–1.00 indicating excellent. T-tests and Pearson correlations were calculated between the demographic variables and the study variables, to identify the control demographic variables for the research model. A multiple linear regression analysis that preceded the model examination revealed several high variance inflation factor (VIF) values, suggesting collinearity between the independent variables (consequences, 2.32; timeline, 2.58; treatment control, 2.43; concern, 2.82; and emotional representations, 2.74). High correlations were found between timeline and lack of treatment control (*r* = .69, *p* < .001), and between consequences, concern, and emotional representations (*r* = .52 to *r* = .68, *p* < .001). Two corresponding aggregate variables were computed with variable means. The research model was examined with path analysis, using AMOS ver 29. Chi square, normed fit index (*NFI)*, non-normed fit index (*NNFI)*, comparative fit index (*CFI)*, and root mean square error of approximation (*RMSEA)* were used as measures of fit. Patient’s age, caregiver’s gender, and patient’s additional chronic illnesses were controlled for. Control variables were correlated among themselves, as were the independent variables and the two mediators. Mediation was examined within the path analysis, with bootstrapping of 5,000 samples and a bias-corrected 95% confidence interval. All variables were standardised.

**Table 1.** Participants’ and patients’ characteristics (*N* = 134).

Sociodemographic characteristics	
**Mean age** participant (SD), range	51.54 (13.51), 24–85
**Mean age** patient (SD), range	71.52 (13.19), 30–97
**Mean length** of ventilation (SD), range, months	9.88 (10.68), 0.25–42
**Gender** participant *n* (%)	
Male	46 (34.3)
Female	88 (65.7)
**Gender** patient *n* (%)	
Male	60 (44.8)
Female	74 (55.2)
**Marital status** participant *n* (%)	
Single	7 (5.2)
Married, in a relationship	94 (70.2)
Divorced	33 (24.6)
**Religiousness** participant *n* (%)	
Secular	81 (60.4)
Traditional	36 (26.9)
Religious	17 (12.7)
**Education** participant *n* (%)	
High school	48 (35.8)
Tertiary education	46 (34.3)
College/university	40 (29.9)
**Kinship to ventilated family member** *n* (%)	
Daughter/son	101 (75.4)
Wife/husband/partner	33 (24.6)
**Living with** the ventilated person prior to hospitalisation *n* (%)	
Yes	56 (41.8)
**Additional chronic diseases** of patient, *n* (%)	
Yes	101 (75.4)
**In the process of withdrawal** from ventilation, *n* (%)	32 (23.9)

SD = Standard deviation.

## Results

As shown in Table 1, about 55% of the patients were female, with a mean age of 71.5 years (*SD* = 13.19, range 30–97 years). The caregivers were mostly female (about 66%); were patients’ children (about 75%) or patients’ spouses (about 25%); and had a mean age of 51.5 years (*SD* = 13.51, range 24–85 years). About 42% of the participants lived with the ventilated person prior to hospitalisation. Most participants were married or in a relationship (about 70%), secular (about 60%), with varying levels of education. Most patients had additional chronic diseases (about 75%), and about a quarter were in the process of withdrawal from ventilation (about 24%).

On average, consequences, symptom burden, and illness understanding were relatively high (all in the upper third of the possible range); timeline, treatment control, concern, and emotional representations were moderate (in the middle third); and self-control was low (in the lower third). Additionally, on average, a sense of coherence, perceived social support, and SWB were relatively high (all in the upper third of the possible range).

**Table 2.** Means, SDs, and correlations for the study variables (*N* = 134).

Variables	1	2	3	4	5	6	7	8	9	10	11
**1. SWB**	1										
**Illness representations**											
2. Consequences	−.02	1									
3. Timeline	−.18*	−.28**	1								
4. Self-control	−.30***	.15	−.38***	1							
5. Treatment control	.32***	.28**	−.69***	.18*	1						
6. Symptom burden	−.25**	.02	.27**	.01	−.25**	1					
7. Concern	−.24**	.63***	−.12	.18*	.04	.23**	1				
8. Understanding	.15	−.09	.21*	−.26**	−.24**	.02	−.06	1			
9. Emotional representations	−.24**	.52***	.03	.25**	−.03	.26**	.68***	−.18*	1		
**10. Sense of coherence**	.57***	−.34***	−.05	−.29***	.17	−.23**	−.52***	.10	−.60***	1	
**11. Perceived social support**	.50***	−.15	−.12	−.32***	.13	−.16	−.14	.16	−.24**	.49***	1
**Mean**	5.32	7.83	6.77	1.04	5.60	8.09	6.49	8.28	5.81	5.87	6.48
**SD**	0.62	2.01	2.28	1.74	3.04	1.97	2.08	1.52	1.88	0.82	0.66
**Possible range**	1–6	0–10	0–10	0–10	0–10	0–10	0–10	0–10	0–10	1–7	1–7
**Actual range**	2.43–6.00	.00–10.00	2.00–9.00	.00–8.00	.00–9.00	2.00–10.00	2.00–9.00	3.00–10.00	.00–9.00	3.36–7.00	3.50–7.00

*p < .05, **p < .01, ***p < .001.

As shown in Table 2, significant associations were found among the study variables. Generally, more severe consequences, higher concern, and greater emotional representations were positively associated with each other (*r* = .52 to *r* = .68, *p* < .001). Likewise, longer timeline, lower self-control, lower treatment control, and higher symptom burden were associated with each other (*r* = −.69 *p* < .001 to *r* = .27, *p* = .002). In addition, lower self-control was associated with higher illness understanding (*r* = −.26, *p* = .003) and lower emotional representations (*r* = .25, *p* = .004); lower treatment control was associated with higher illness understanding (*r* = −.26, *p* = .005); and higher symptom burden was associated with higher concern (*r* = .23, *p* = .007) and emotional representations (*r* = .26, *p* = .003).

Less severe consequences, lower self-control, lower symptom burden, lower concern, and lower emotional representations were associated with a higher sense of coherence (*r* = −.23 *p* = .008 to *r* = −.60, *p* < .001). Lower self-control and lower emotional representations were associated with higher perceived social support (*r* = −.24 *p* = .006 to *r* = −.32, *p* < .001). Perceived shorter timeline, lower self-control, higher treatment control, lower symptom burden, lower concern, and lower emotional representations were associated with higher SWB (*r* = −.30 to *r* = .32, *p* < .001). Finally, sense of coherence, perceived social support, and SWB were positively associated with each other (*r* = .49 to *r* = .57, *p* < .001).

Several demographic variables were found to have significant associations with the study variables. First, caregiver’s gender was associated with SWB, such that it was higher for women (*mean* = 5.40, *SD* = 0.55) than men (*mean* = 5.17, *SD* = 0.71), *t*(132) = 2.01, *p* = .046. Consequences and emotional representations were perceived as more severe by women (*mean* = 8.11, *SD* = 1.64 and *mean* = 6.18, *SD* = 1.69, respectively) than by men (*mean* = 7.28, *SD* = 2.50 and *mean* = 5.11, *SD* = 2.04, respectively), *t*(65.87) = 2.04, *p* = .046 and *t*(132) = 3.25, *p* = .001, respectively.

Second, the presence of patient’s additional chronic illnesses was associated with SWB and perceived social support, such that these were higher among caregivers of patients with no additional chronic illnesses (*mean* = 5.42, *SD* = 0.80 and *mean* = 6.72, *SD* = 0.51, respectively) than among caregivers of patients with additional chronic illnesses (*mean* = 5.29, *SD* = 0.54 and *mean* = 6.40, *SD* = 0.69, respectively), *t*(44.12) = 2.41, *p* = .010 and *t*(70.68) = 3.47, *p* < .001, respectively. In addition, timeline, lack of treatment control, and symptom burden were perceived as more severe by caregivers of patients with additional chronic illnesses (*mean* = 7.13, *SD* = 2.18, *mean* = 6.82, *SD* = 2.76, and *mean* = 8.45, *SD* = 1.79, respectively) than among caregivers of patients with no additional chronic illnesses (*mean* = 5.67, *SD* = 2.25, *mean* = 5.21, *SD* = 3.04, and *mean* = 7.00, *SD* = 2.11, respectively), *t*(132) = 3.32, *p* = .001, *t*(132) = 2.70, *p* = .008, and *t*(132) = 3.85, *p* < .001, respectively.

Third, higher patient’s age was associated with less severe consequences (*r* = −.42, *p* < .001), longer timeline (*r* = .42, *p* < .001), less treatment control (*r* = −.49, *p* < .001), lower concern (*r* = −.20, *p* = .021), and higher illness understanding (*r* = .24, *p* = .005).

It should be noted, as expected, that patient’s age was positively associated with caregiver’s age (*r* = .57, *p* < .001), and that patients who were cared for by their children were, on average, older than patients who were cared for by their spouses (*mean* = 74.70, *SD* = 11.27, *mean* = 61.79, *SD* = 14.04, *t*(132) = 5.37, *p* < .001). Thus, controlling for patient’s age was, to some extent, indirectly controlling for caregiver’s age and kinship. Quite similarly, patient’s age was, to some extent, indirectly controlling for caregiver’s living with/not living with patient (prior to hospitalisation).

Several other demographic and background variables (e.g. patient’s gender, length of ventilation) were associated with the study variables, yet to a lesser extent than patient’s age, caregiver’s gender, and additional chronic illnesses. These other variables were not included in the model analysis due to sample size. Thus, the study model was examined using patient’s age, caregiver’s gender (1-male, 0-female), and additional chronic illnesses (1-yes, 0-no) as control variables.

### The study model

**Figure 1.**
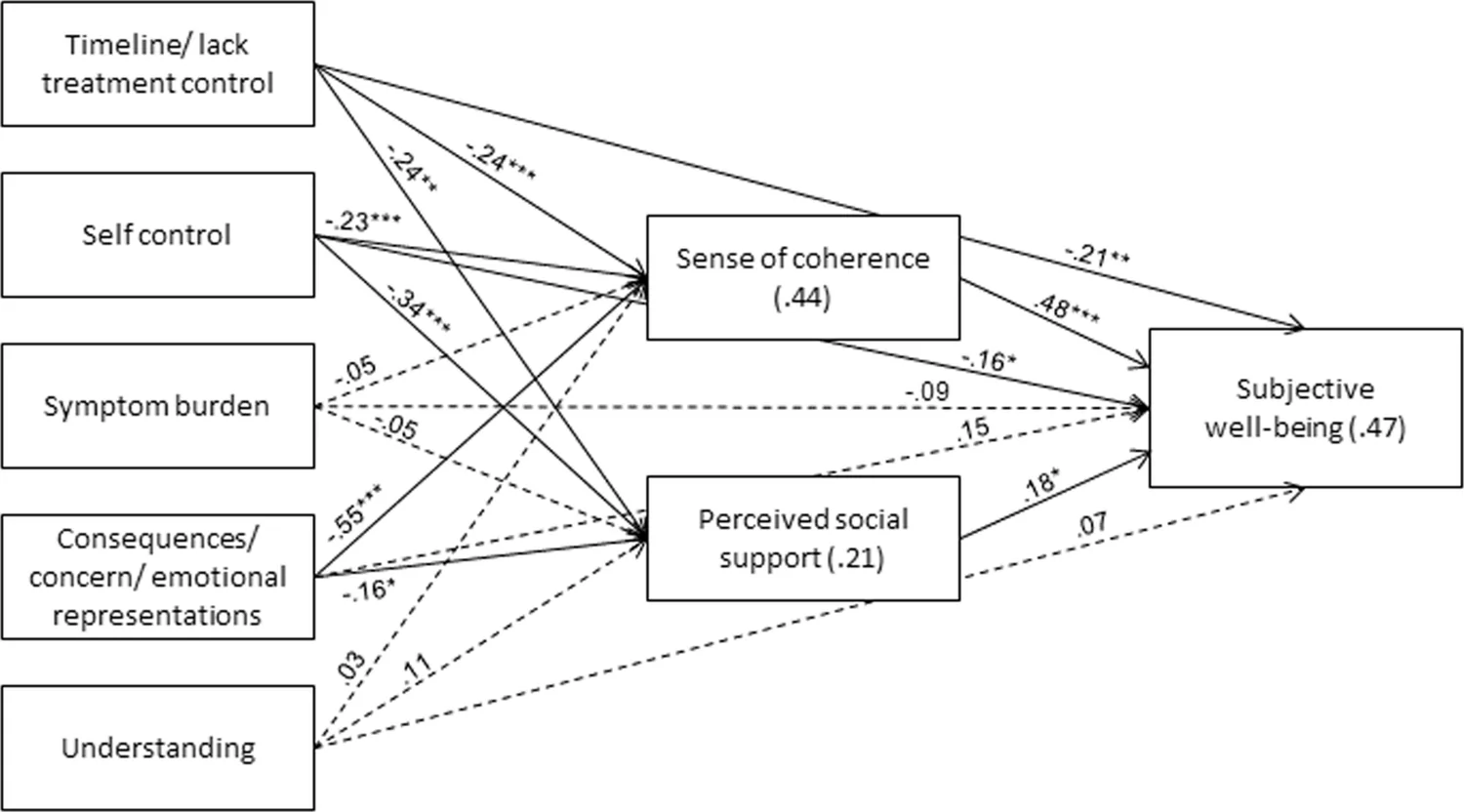
Path analysis for subjective well-being, illness representations, sense of coherence, and perceived social support, among family members of hospitalised ventilated patients. Note: R^2^—values within rectangles. *β*—standardised regression coefficients- values above one-way arrows. Straight lines- significant associations, dashed lines- non significant associations. *p < .05, **p < .01, ***p < .001.

The study model was examined with path analysis, using AMOS ver 29. Patient’s age, caregiver’s gender, and patient’s additional chronic illnesses were controlled for. Control variables were correlated among themselves, as were the independent variables and the two mediators. All variables were standardised. The model was found to fit the data: χ^2^(16) = 16.50, *p* = .419, NFI = .956, NNFI = .995, CFI = .998, RMSEA = .015. Figure 1 presents the results of the path analysis.

Results show that the perception of a shorter timeline with higher treatment control, lower self-control, and the perceptions of lower consequences, lower concern, and lower emotional representations were associated with both a higher sense of coherence (*β* = −.23 to *β* = −.55, *p* < .001) and higher perceived social support (*β* = −.16 *p* = .043 to *β* = −.34, *p* < .001). Both a higher sense of coherence and higher perceived social support were associated with higher SWB (*β* = .18 *p* = .017 and *β* = .48, *p* < .001, respectively). Symptom burden and illness understanding were not associated with sense of coherence, perceived social support, or SWB. In light of these associations, several indirect effects were found significant, as shown in Table 3.

The perception of a shorter timeline with higher treatment control, lower self-control, and the perceptions of lower consequences, lower concern, and lower emotional representations were associated with a higher sense of coherence and higher perceived social support, which in turn were associated with higher SWB (95%CI for sense of coherence = −0.22 to −0.05, −0.20 to −0.04, and −0.39 to −0.15, respectively; 95%CI for perceived social support = −0.11 to −0.01, −0.14 to −0.01, and −0.09 to −0.01, respectively). That is, a shorter timeline with higher treatment control, lower self-control, and lower consequences, lower concern, and lower emotional representations were indirectly associated with higher SWB, through higher sense of coherence and perceived social support. Symptom burden and illness understanding were not associated with SWB, directly or indirectly.

## Discussion

This study offers important insights into the relationship between illness representations, coping resources, and SWB among family caregivers of patients who require PMV. This population represents a uniquely vulnerable group due to the intensive demands of managing a patient reliant on life-sustaining technology and facing a poor prognosis (Milton et al., 2022). These challenges create a distinctive caregiving context in which psychological, emotional, and social factors interact in intricate ways to shape caregivers' well-being.

The association between caregivers' perceptions of shorter illness timelines, higher treatment control, and better SWB underscores the important role of optimistic illness representations in fostering resilience (Shinan-Altman & Becker, 2024). In the PMV context, caregivers often face a condition that is both chronic and life-threatening, characterised by high levels of uncertainty regarding outcomes (Jang & Wang, 2020; Trudzinski et al., 2022). When caregivers perceive the situation as more manageable or time-limited, this perception may mitigate the psychological toll of prolonged caregiving by fostering a sense of hope and control. This finding supports theories of cognitive appraisal, which posit that individuals' evaluations of stressors are associated with the ability to cope (Foster & Chaboyer, 2018; Lazarus, 1984). Importantly, the perception of treatment control may be especially salient in the PMV context, as caregivers' involvement in day-to-day decisions—such as monitoring the ventilator or coordinating care—can reinforce a sense of efficacy and empowerment.

Conversely, in the current study, negative illness representations such as perceptions of severe consequences and heightened emotional responses were associated with lower SWB, alongside lower levels of sense of coherence and perceived social support. These findings align with research showing that threatening illness perceptions amplify stress and reduce caregivers’ capacity to engage with coping resources (Liu et al., 2021; Lo Sterzo & Orgeta, 2017). In the PMV setting, where caregivers often witness their loved ones' significant functional decline and dependence on mechanical support, such perceptions may trigger chronic stress responses, including feelings of helplessness and anticipatory grief. The intense emotional toll associated with these representations may also erode caregivers' ability to seek or accept social support, further isolating them during a crucial time (Muñoz-Bermejo et al., 2020).

The findings highlight the mediating roles of sense of coherence and perceived social support as key factors linking illness representations to SWB. Sense of coherence reflects an individual’s ability to view life as comprehensible, manageable, and meaningful (Antonovsky, 1987). In the PMV caregiving context, this resource is essential for navigating the complexities of medical care, from understanding intricate ventilator settings to managing frequent hospitalisations. Caregivers with a strong sense of coherence may be better equipped to integrate the caregiving role into their broader life narrative, finding purpose even amidst significant challenges (Del-Pino-Casado et al., 2019). The study’s findings suggest that greater alignment between illness representations and caregivers’ ability to make sense of their circumstances is associated with higher sense of coherence, as well as greater psychological resilience and well-being.

Perceived social support emerged as an important external resource that mediated the relationship between illness representations and SWB. The PMV caregiving context often isolates caregivers due to the intensive time commitment and emotional strain involved in providing care (Marcus et al., 2021). Social networks—comprising family, friends, and healthcare providers—offer practical assistance, emotional validation, and a sense of connection that can alleviate this isolation (Leung et al., 2020). However, the relationship between illness representations and social support can be intricate. Caregivers who hold more negative views of the illness might reduce the quality or availability of support by withdrawing from their social circles or expressing distress in ways that may overwhelm those offering help (Kalolo et al., 2023; Taylor, 2011). By contrast, caregivers with more positive or adaptive views of the illness are more likely to actively seek out and effectively use support, which contributes to their overall well-being.

The findings indicate that symptom burden and illness understanding were not significantly associated with coping resources or SWB. In other caregiving contexts, these factors are often significant predictors of caregiver outcomes (Chu et al., 2022; Cipolletta et al., 2020). However, in the PMV setting, the highly technical and dependent nature of the condition may reduce the perceived relevance of symptom-specific knowledge or management to caregivers' broader sense of well-being. This finding raises questions about the unique stressors in the PMV caregiving context and suggests that traditional models of caregiver burden such as the Stress Process Model (Pearlin et al., 1990) may require adaptation for this population. For example, caregivers may prioritise their emotional resilience or social connections over gaining detailed medical knowledge, particularly if healthcare providers play a primary role in symptom management.

In this study, female caregivers reported more severe emotional representations and consequences, yet achieved higher SWB than did their male counterparts. This finding may reflect gender differences in caregiving roles and socialisation. Women are often socialised to provide emotional labour and maintain interpersonal relationships, which may make them more attuned to the emotional toll of caregiving (Bueno & Chase, 2023) but also more adept at mobilising social support networks (Rodríguez-Madrid et al., 2019). In contrast, male caregivers may experience greater challenges in navigating the relational aspects of caregiving, potentially limiting their access to supportive resources (Lopez–Anuarbe & Kohli, 2019). These findings highlight the importance of gender-sensitive interventions that address the distinct needs and strengths of male and female caregivers in the PMV context.

In the current study, caregivers of patients with multiple health conditions reported lower SWB, reflecting the heightened demands of managing comorbidities alongside mechanical ventilation. This finding aligns with the cumulative burden theory, which posits that the accumulation of stressors exacerbates psychological strain (Jacobs et al., 2021). For these caregivers, the need for integrated care models that address both patient and caregiver needs is particularly critical. Such models could involve coordinated care planning, regular mental health assessments, and the provision of respite services to alleviate caregiver burden.

The current study has several limitations. Its cross-sectional design precludes causal inferences, and longitudinal studies are needed to track changes in illness representations, coping resources, and SWB over time. Most importantly, a longitudinal study is required to assess mediation, as the direction of the associations cannot be determined unequivocally with a cross-sectional design. Theory has supported our model, yet the criticism of mediation models with cross-sectional data is wide (e.g. Li et al., 2023; Preacher, 2015). We therefore recommend to treat the mediation model found in this study as a basis for future longitudinal studies. The relatively small sample size and recruitment through online platforms may limit the generalisability of the findings. Future research should aim to include larger and more diverse populations, particularly caregivers in different healthcare and cultural settings. Additionally, reliance on self-reported data introduces potential biases; employing mixed methods could provide a more comprehensive understanding of caregivers’ experiences.

**Table 3.** Indirect effects for SWB with illness representations, sense of coherence, and perceived social support, among family members of hospitalised ventilated patients (*N* = 134).

Predictor	Mediator	Effect (*SE*)	*p*	95%*CI*
Timeline/lack of treatment control	Sense of coherence	−0.12 (0.04)	**<.001**	−0.22, −0.05
Self-control		−0.11 (0.04)	**.002**	−0.20, −0.04
Symptom burden		−0.02 (0.03)	.469	−0.10, 0.04
Consequences/concern/emotional representations		−0.26 (0.06)	**<.001**	−0.39, −0.15
Illness understanding		0.02 (0.03)	.582	−0.05, 0.09
Timeline/lack of treatment control	Perceived social support	−0.04 (0.03)	**.016**	−0.11, −0.01
Self-control		−0.06 (0.03)	**.016**	−0.14, −0.01
Symptom burden		−0.01 (0.02)	.411	−0.05, 0.02
Consequences/concern/emotional representations		−0.03 (0.02)	**.036**	−0.09, −0.01
Illness understanding		0.02 (0.02)	.097	−0.01, 0.07

This study contributes to theoretical advancements by extending the SRM to the PMV caregiving context, emphasising the mediating roles of sense of coherence and perceived social support. The findings suggest that the SRM’s focus on illness representations could be enriched by considering the dynamic interplay between cognitive appraisals, emotional responses, and resource utilisation. The non-significant role of symptom burden and illness understanding challenges traditional assumptions and invites further exploration of how these dimensions operate in highly specialised caregiving contexts.

Practically, the findings underscore the need for interventions tailored to the unique demands of PMV caregiving. Psychoeducational programmes that help caregivers reframe maladaptive illness representations—such as emphasising treatment control and manageability—could enhance their sense of coherence and well-being. Structured support groups specifically designed for PMV caregivers may provide opportunities for shared learning, emotional validation, and problem-solving. Additionally, healthcare providers should adopt family-centred care models that prioritise clear communication, empathy, and the proactive inclusion of caregivers in decision-making processes. These recommendations serve as a guiding framework for practitioners, programme developers, organisational leaders, and policymakers to design and implement interventions that address illness perceptions, strengthen coping resources such as sense of coherence and social support, and enhance the overall well-being of family caregivers of patients receiving PMV.

## Supplementary Material

Supplementary Material Supplementary Material.xlsx

## Data Availability

The datasets generated and analysed during the current study are available from the corresponding author upon reasonable request.
